# Targeted next-generation sequencing for cancer-associated gene mutation and copy number detection in 206 patients with non–small-cell lung cancer

**DOI:** 10.1080/21655979.2021.1890382

**Published:** 2021-02-25

**Authors:** Songbai Zheng, Xiaodan Wang, Ying Fu, Beibei Li, Jianhua Xu, Haifang Wang, Zhen Huang, Hui Xu, Yurong Qiu, Yaozhou Shi, Kui Li

**Affiliations:** a Translational Medicine Research Institute, Guangzhou Huayin Medical Laboratory Center Co., Ltd., Guangzhou, China; b Shenzhen Institute of Synthetic Biology, Shenzhen Institutes of Advanced Technology, Chinese Academy of Sciences, Shenzhen, China; c CAS Key Laboratory of Quantitative Engineering Biology, Shenzhen Institutes of Advanced Technology, Chinese Academy of Sciences, Shenzhen, China; d Research and Development Institute, Sinotech Genomics, Shanghai, China; e Laboratory Medicine Center, Shunde Hospital of Guangzhou University of Chinese Medicine, Guangzhou, China; f Laboratory Medicine Center, The Second Clinical Medical College of Guangzhou University of Chinese Medicine, Guangzhou, China; g Laboratory Medicine Center, Nanfang Hospital, Southern Medical University, Guangzhou, China; h Technical Service Department, Guangzhou Huayin Medical Research Institute Co., Ltd., Guangzhou, China

**Keywords:** sequencing, panel, lung, cancer, mutation

## Abstract

The knowledge of genetic variation in Chinese patients with non–small-cell lung cancer (NSCLC) is still limited. We aimed to profile this genetic variation in 206 Chinese patients with NSCLC using next-generation sequencing. Tumor tissues or whole-blood samples were collected and subjected to whole-exome targeted next-generation sequencing, which included 565 tumor-associated genes, for somatic gene mutation screening and copy number variation (CNV) detection. Potential functions of most commonly mutated genes and genes with CNV were predicted by Gene Ontology (GO) and Kyoto Encyclopedia of Genes and Genomes (KEGG) analyses. Atotal of 18,749 mutations were identified using targeted next-generation sequencing, and 85.3% of them were missense mutations. Among the mutation, conversions between pyrimidine and purine were predominant, and C> T/G > A was the most common substitution type. High frequencies of mutations were noted in TP53 (47.6%), EGFR (41.7%), CREBBP (23.1%), KMT2C (16.9%), MUC2 (16.6%), DNMT3A (15.5%), LRP1B (15.5%), MUC4 (15.5%), CDC27 (15.2%), and KRAS (12.8%). EGFR and KRAS mutations were mutually exclusive. The tumor mutation load showed differences depending on gender and tumor type. CNV analysis showed that BCORL1 and ARAF have the highest copy number amplification, whereas KDM6A and RBM10 showed the highest copy number deletion. GO and KEGG analyses indicated that high-frequency mutations and CNV genes were concentrated in tumor-related PI3K-Akt, FoxO, and Ras signaling pathway. Cumulatively, we studied somatic gene mutations involved in NSCLC and predicted their clinical significance in Chinese population. These findings may provide clues for etiology and drug target of NSCLC.

## GRAPHICAL ABSTRACT

**Figure:**
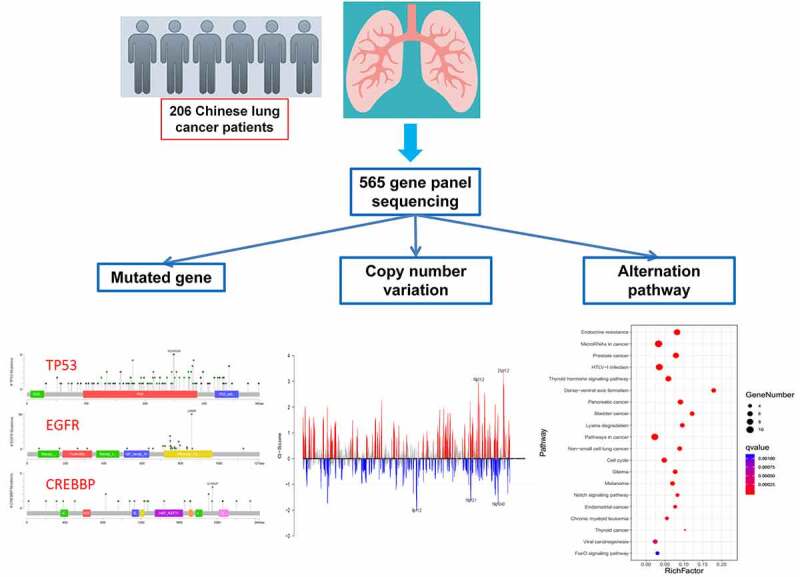

## Introduction

Lung cancer has become the leading deadly malignancy in China and globally, in both men and women [1]. According to 2015 statistics, there were approximately 730,000 new cases of lung cancer in China and more than 430,000 people died from this disease. Lung cancer is divided into non–small-cell lung carcinoma (NSCLC) and small-cell lung carcinoma (SCLC) [2], with NSCLC accounting for more than 85% of cases [3]. Moreover, NSCLC has a high mortality rate. Despite extensive research on different treatment options, patients diagnosed with NSCLC (all stages) have a mortality rate of more than 50% within 1 year and an overall 5-year survival rate of less than 18% [4]. These data suggest that there is still a need for new targeted therapeutic drug research of NSCLC, and analyses of the underlying mechanism of NSCLC from a genetic level may provide clues for finding new therapeutic targets.

Next-generation sequencing (NGS) is an approach widely used for the characterization of genetic features. Using an NGS platform, whole-genome sequencing, whole-exome sequencing, whole-transcriptome sequencing, and targeted sequencing can be performed for multiple specific genomic regions. It is a high-throughput and economical method for detecting multiple genetic variations [5]. Many studies have used NGS to analyze genetic variation, tumor mutation burden, and microsatellite instability in solid tumors such as colorectal cancer, gastric cancer, and breast cancer [6,7]. Target sequencing is also used for the identification of variations in genes causing lung cancer. Based on these NGS data, several important genes related to lung cancer have been identified, for exampletumor protein P53 (TP53), phosphatase and tensin homolog (PTEN), epidermal growth factor receptor (EGFR), KRAS proto-oncogene, GTPase (KRAS), neurofibromin 1 (NF1), ATM serine/threonine kinase (ATM), phosphatidylinositol-4,5-bisphosphate 3-kinase catalytic subunit alpha (PIK3CA), and fibroblast growth factor receptor 4 (FGFR4) [8–13]. However, the knowledge of genetic variation in NSCLC remains limited in the Chinese population. Existing studies have focused on a small range of genes. For example, Wen et al. performed NGS of 37 cancer-related genes and selected introns of eight genes [14]. Tsoulos et al. focused on a custom panel comprising 23 genes [13,15]. Therefore, a broader panel containing NSCLC-related genes of great significance for the diagnosis and precise treatment of NSCLC is still needed.

Here, we established a panel to detect somatic mutations in 206 samples from Chinese patients. To include as many NSCLC-related genes as possible, the panel comprised 565 genes that were associated with sensitivity and side effects of commonly used chemotherapeutic drugs in clinic and cancer risk. Our study expected to provide an overview of the characteristics of tumor genetic variation in Chinese patients with NSCLC, and provide clues for the clinical diagnosis to enable individualized therapy and find new therapeutic targets of NSCLC.

## Materials and methods

### Patient and DNA isolation

Surgically resected tumor tissues or venous blood samples were collected from 206 NSCLC patients. Genomic DNA was isolated from tissues or blood using the QIAGEN DNeasy Blood & Tissue Kit (#69504, Qiagen, Germany). All patients gave written informed consent to participate in this study.

### Whole-exome next-generation and targeted gene sequencing

DNA libraries for whole-exome NGS were prepared using NEBNext® Ultra™ DNA Library Prep Kit (NEB #E7645, NEB, USA) for Illumina, in accordance with the manufacturer’s instructions. Whole-exome capture was performed using TruSeq Exome Enrichment kit (Illumina # 20020183, USA). For targeted gene sequencing, a panel comprising 565 tumor-related genes was prepared. Targeted genes were enriched with the TruSeq Custom Enrichment kits (Illumina). Samples were sequencing using the HiSeq X TEN platform (Illumina).

### Bioinformatic analysis

The adapter sequence in the raw data was removed by cutadapt, after which high-quality reads were aligned to the human reference genome (hg19) using BWA [16] with the default parameters. Somatic mutations were detected by MuTect [17] based on the alignment. Somatic SNVs with high confidence were called if the following criteria were met: (I) both tumor and normal samples should have coverage of ≥10× at the genomic position; and (II) the variants should be supported by at least 5% of the total reads in the tumor. Copy number variation (CNV) for each tumor sample was determined by ADTEx [18]. Gene Ontology (GO) and Kyoto Encyclopedia of Genes and Genomes (KEGG) pathway enrichment analyses of mutated genes were performed using KOBAS [19]. Enriched terms were defined as those with FDR of <0.01.

### Statistical analysis

The difference in Tumor mutation burden (TMB) between male and female and adenocarcinoma and squamous carcinoma were analyzed using Student’s t-test method. Correlation between TMB and age were analyzed using Pearson Correlation Coefficient method.

## Results

Analyses of the underlying mechanism of NSCLC from a genetic level may provide clues for studying new therapeutic targets for drugs in NSCLC treatment; however, the knowledge of the genetic variation of NSCLC remains limited in Chinese population. Moreover, NGS is a widely used approach for the characterization of genetic characteristics. In this study, we established a panel containing 565 genes that were associated with sensitivity and side effects of commonly used chemotherapeutic drugs in clinic and cancer risk to detect somatic mutations in samples from 206 Chinese patients. A total of 18,749 mutations were identified using targeted NGS and 85.3% of them were missense mutations. Among the mutations, conversions between pyrimidine and purine were dominant, and C > T/G > A was the most common substitution type. High frequencies of mutations were noted in TP53 (47.6%), EGFR (41.7%), CREB binding protein (CREBBP) (23.1%), lysine methyltransferase 2 C (KMT2C) (16.9%), Mucin 2 (MUC2) (16.6%), DNA methyltransferase 3 alpha (DNMT3A) (15.5%), LDL receptor related protein 1B (LRP1B) (15.5%), Mucin 4 (MUC4) (15.5%), cell division cycle 27 (CDC27 (15.2%), and KRAS (12.8%). EGFR and KRAS mutations were mutually exclusive. The tumor mutation load showed BCL6 corepressor like 1 (BCORL1) and a-raf proto-oncogene (ARAF) have the highest copy number amplification, whereas lysine demethylase 6A (KDM6A) and RNA binding motif protein 10 (RBM10) showed the highest copy number deletion. GO and KEGG analyses indicated that high-frequency mutations and CNV genes were concentrated in the tumor-related PI3K-Akt, FoxO, and Ras signaling pathway.

### Overview of somatic mutation in patients with NSCLC

**Figure 1.**
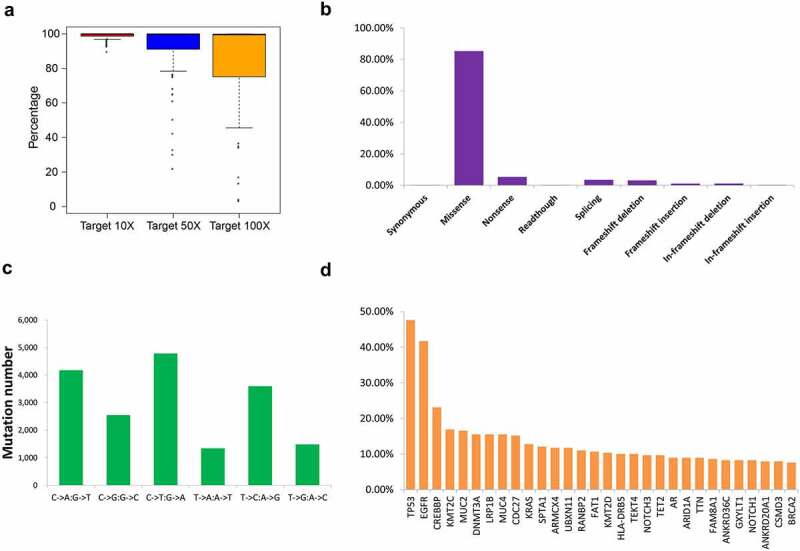
Overview of the mutation status of the 206 patients with NSCLC based on next-generation sequencing. (a) Coverage depth for gene regions. Distribution of gene mutation types (b) and single mutation types (c) in the 206 patients with NSCLC. (d) Schematic showing 30 genes with the highest mutation frequency

To obtain an overview of somatic mutation in Chinese patients with NSCLC patients, we recruited 206 Chinese patients with NSCLC and performed targeted NGS. The mean age of the 206 enrolled patients with NSCLC was 65 years (range 54–86). Of these, 81 (39.3%) were male and 125 (60.7%) were female. Individual clinical information is listed in Table 1. To obtain the somatic mutation spectrum of the 206 patients, next-generation sequencing-based technology was used to capture 565 genes from tumor tissues and peripheral blood of patients with NSCLC. As shown in Figure 1(a), the coverage depth of the captured regions of most genes was at least 50×, with an average coverage depth of 914× (Table 1) (Figure 1(a)).

**Table 1.** Clinical information of the 206 NSCLC patients

SampleID	Gender	Age	Clinical diagnosis
P1	Female	63	Non-small cell lung cancer
P2	Male	65	Adenocarcinoma
P3	Male	79	Adenocarcinoma
P4	Male	54	Adenocarcinoma
P5	Female	68	Adenocarcinoma
P6	Male	71	Squamous
P7	Female	63	Squamous
P8	Male	72	Squamous
P9	Male	63	Squamous
P10	Male	54	Squamous
P11	Female	74	Non-small-cell lung cancer
P12	Male	59	Non-small-cell lung cancer
P13	Male	69	Non-small-cell lung cancer
P14	Male	44	Non-small-cell lung cancer
P15	Male	68	Adenocarcinoma
P16	Female	49	Non-small-cell lung cancer
P17	Male	57	Adenocarcinoma
P18	Female	61	Non-small-cell lung cancer
P19	Male	56	Squamous
P20	Male	65	Adenocarcinoma
P21	Male	63	Squamous
P22	Female	64	Non-small-cell lung cancer
P23	Female	57	Non-small-cell lung cancer
P24	Female	45	Adenocarcinoma
P25	Female	51	Adenocarcinoma
P26	Female	50	Adenocarcinoma
P27	Male	82	Non-small-cell lung cancer
P28	Male	56	Non-small-cell lung cancer
P29	Female	64	Adenocarcinoma
P30	Male	71	Non-small-cell lung cancer
P31	Male	46	Adenocarcinoma
P32	Male	52	Squamous
P33	Female	48	Non-small-cell lung cancer
P34	Male	61	Non-small-cell lung cancer
P35	Male	35	Squamous
P36	Male	69	Small cell lung cancer
P37	Female	69	Non-small-cell lung cancer
P38	Male	64	Non-small-cell lung cancer
P39	Male	65	Non-small-cell lung cancer
P40	Female	75	Non-small-cell lung cancer
P41	Female	58	Adenocarcinoma
P42	Female	38	Adenocarcinoma
P43	Female	63	Non-small-cell lung cancer
P44	Male	62	Non-small-cell lung cancer
P45	Male	79	Non-small-cell lung cancer
P46	Male	51	Non-small-cell lung cancer
P47	Female	60	Adenocarcinoma
P48	Male	62	Non-small-cell lung cancer
P49	Male	68	Non-small-cell lung cancer
P50	Female	69	Non-small-cell lung cancer
P51	Female	53	Non-small-cell lung cancer
P52	Female	57	Adenocarcinoma
P53	Female	61	Adenocarcinoma
P54	Male	58	Non-small-cell lung cancer
P55	Male	54	Neuroendocrine
P56	Female	77	Non-small-cell lung cancer
P57	Female	35	Non-small-cell lung cancer
P58	Female	70	Adenocarcinoma
P59	Male	79	Non-small-cell lung cancer
P60	Male	66	Non-small-cell lung cancer
P61	Male	68	Non-small-cell lung cancer
P62	Male	68	Non-small-cell lung cancer
P63	Male	61	Non-small-cell lung cancer
P64	Male	80	Non-small-cell lung cancer
P65	Male	70	Non-small-cell lung cancer
P66	Female	39	Adenocarcinoma
P67	Female	50	Adenocarcinoma
P68	Male	67	Non-small-cell lung cancer
P69	Male	49	Non-small-cell lung cancer
P70	Male	72	Adenocarcinoma
P71	Male	54	Non-small-cell lung cancer
P72	Male	52	Adenocarcinoma
P73	Female	68	Non-small-cell lung cancer
P74	Female	73	Non-small-cell lung cancer
P75	Male	69	Adenocarcinoma
P76	Female	71	Adenocarcinoma
P77	Female	66	Non-small-cell lung cancer
P78	Male	69	Adenocarcinoma
P79	Male	62	Squamous
P80	Male	54	Non-small-cell lung cancer
P81	Female	47	Non-small-cell lung cancer
P82	Male	76	Non-small-cell lung cancer
P83	Male	86	Non-small-cell lung cancer
P84	Male	73	Non-small-cell lung cancer
P85	Male	72	Non-small-cell lung cancer
P86	Male	43	Adenocarcinoma
P87	Female	67	Adenocarcinoma
P88	Male	55	Non-small-cell lung cancer
P89	Male	77	Small cell lung cancer
P90	Male	57	Non-small-cell lung cancer
P91	Female	54	Adenocarcinoma
P92	Male	65	Neuroendocrine
P93	Female	72	Adenocarcinoma
P94	Male	62	Squamous
P95	Female	45	Non-small-cell lung cancer
P96	Female	45	Non-small-cell lung cancer
P97	Female	51	Adenocarcinoma
P98	Female	65	Non-small-cell lung cancer
P99	Male	61	Adenocarcinoma
P100	Male	79	Squamous
P101	Female	64	Adenocarcinoma
P102	Male	75	Non-small-cell lung cancer
P103	Male	67	Adenocarcinoma
P104	Male	72	Non-small-cell lung cancer
P105	Male	79	Adenocarcinoma
P106	Female	51	Non-small-cell lung cancer
P107	Female	78	Non-small-cell lung cancer
P108	Male	58	Non-small-cell lung cancer
P109	Female	69	Adenocarcinoma
P110	Male	82	Non-small-cell lung cancer
P111	Male	76	Non-small-cell lung cancer
P112	Male	61	Adenocarcinoma
P113	Female	64	Adenocarcinoma
P114	Female	69	Non-small-cell lung cancer
P115	Male	85	Adenocarcinoma
P116	Male	56	Non-small-cell lung cancer
P117	Female	62	Non-small-cell lung cancer
P118	Male	62	Squamous
P119	Male	56	Squamous
P120	Male	68	Squamous
P121	Male	63	Adenocarcinoma
P122	Male	58	Non-small-cell lung cancer
P123	Male	64	Adenocarcinoma
P124	Male	68	Non-small-cell lung cancer
P125	Male	59	Adenocarcinoma
P126	Male	67	Non-small-cell lung cancer
P127	Female	78	Non-small-cell lung cancer
P128	Female	66	Non-small-cell lung cancer
P129	Female	67	Non-small-cell lung cancer
P130	Female	57	Non-small-cell lung cancer
P131	Female	74	Non-small-cell lung cancer
P132	Male	55	Non-small-cell lung cancer
P133	Male	62	Squamous
P134	Male	66	Squamous
P135	Female	56	Non-small-cell lung cancer
P136	Male	60	Non-small-cell lung cancer
P137	Male	81	Non-small-cell lung cancer
P138	Male	63	Non-small-cell lung cancer
P139	Female	49	Adenocarcinoma
P140	Male	56	Non-small-cell lung cancer
P141	Male	74	Non-small-cell lung cancer
P142	Female	49	Non-small-cell lung cancer
P143	Male	65	Non-small-cell lung cancer
P144	Female	52	Adenocarcinoma
P145	Male	40	Non-small-cell lung cancer
P146	Male	66	Adenocarcinoma
P147	Female	65	Small cell lung cancer
P148	Female	68	Large cell lung cancer
P149	Male	41	Adenocarcinoma
P150	Male	54	Adenocarcinoma
P151	Female	53	Non-small-cell lung cancer
P152	Male	76	Non-small-cell lung cancer
P153	Female	49	Non-small-cell lung cancer
P154	Female	71	Adenocarcinoma
P155	Male	69	Non-small-cell lung cancer
P156	Male	60	Adenocarcinoma
P157	Male	52	Non-small-cell lung cancer
P158	Female	68	Non-small-cell lung cancer
P159	Male	62	Adenocarcinoma
P160	Male	75	Non-small-cell lung cancer
P161	Male	65	Non-small-cell lung cancer
P162	Male	65	Non-small-cell lung cancer
P163	Male	55	Non-small-cell lung cancer
P164	Male	68	Non-small-cell lung cancer
P165	Male	57	Adenocarcinoma
P166	Female	48	Neuroendocrine
P167	Male	73	Adenocarcinoma
P168	Male	62	Adenocarcinoma
P169	Female	70	Adenocarcinoma
P170	Female	61	Non-small-cell lung cancer
P171	Male	65	Adenocarcinoma
P172	Male	75	Non-small-cell lung cancer
P173	Male	53	Non-small-cell lung cancer
P174	Female	53	Non-small-cell lung cancer
P175	Male	75	Adenocarcinoma
P176	Male	40	Non-small-cell lung cancer
P177	Male	65	Non-small-cell lung cancer
P178	Female	67	Non-small-cell lung cancer
P179	Male	70	Non-small-cell lung cancer
P180	Male	55	Non-small-cell lung cancer
P181	Female	68	Small cell lung cancer
P182	Male	56	Adenocarcinoma
P183	Male	66	Non-small-cell lung cancer
P184	Female	70	Non-small-cell lung cancer
P185	Male	62	Squamous
P186	Female	55	Adenocarcinoma
P187	Female	71	Adenocarcinoma
P188	Female	63	Adenocarcinoma
P189	Female	69	Non-small-cell lung cancer
P190	Female	51	Adenocarcinoma
P191	Female	46	Adenocarcinoma
P192	Female	74	Non-small-cell lung cancer
P193	Female	61	Non-small-cell lung cancer
P194	Male	47	Non-small-cell lung cancer
P195	Male	68	Squamous
P196	Male	49	Non-small-cell lung cancer
P197	Female	83	Non-small-cell lung cancer
P198	Female	66	Adenocarcinoma
P199	Male	56	Squamous
P200	Male	54	Non-small-cell lung cancer
P201	Male	51	Non-small-cell lung cancer
P202	Female	70	Adenocarcinoma
P203	Female	62	Non-small-cell lung cancer
P204	Male	66	Adenocarcinoma
P205	Male	56	Non-small-cell lung cancer
P206	Male	63	Adenocarcinoma

A total of 18,749 mutations were identified, and the dominant mutation type was missense mutation (85.3%) (Figure 1(b), Table 2). Single-mutation variation analysis revealed that the dominant base mutations predominantly involved purines (Figure 1(c)) and that C > T/G > A was the most common substitution type. Of the mutated genes, 79 had a mutation frequency of more than 5%. Among these, the top ten most frequently mutated genes were TP53 (47.6%), EGFR (41.7%), CREBBP (23.1%), KMT2C (16.9%), MUC2 (16.6%), DNMT3A (15.5%), LRP1B (15.5%), MUC4 (15.5%), CDC27 (15.2%), and KRAS (12.8%) (Figure 1(d)).

**Table 2.** Mutation information of the 206 NSCLC patients

Type	Number	Percentage
Synonymous	32	0.17%
Missense	15,985	85.26%
Nonsense	996	5.31%
Readthough	38	0.20%
Splicing	653	3.48%
Frameshift deletion	585	3.12%
Frameshift insertion	199	1.06%
In-frameshift deletion	214	1.14%
In-frameshift insertion	47	0.25%
Total	18,749	100.00%

### TMB analysis in patients with NSCLC

**Figure 2.**
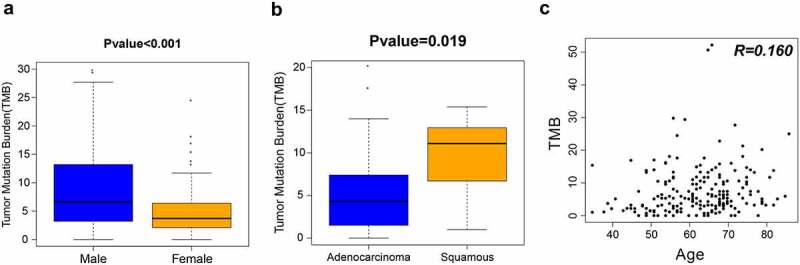
TMB analysis of 206 patients with NSCLC. (a) Differences in TMB by sex. (b) Differences in TMB by tumor type. (c) Correlation between TMB and age. TMB, tumor mutation burden

TMB has been proved to be an immunotherapy biomarker in clinical oncology, including NSCLC. To explore the association between TMB and NSCLC in Chinese patients, we performed comparative analysis of the sexes and different tumor subtypes showed that TMB in females was lower than that in males (Figure 2(a)). The median TMB for men is 6.6 Mutations/Mb, and the median TMB for women is 3.7 Mutations/Mb. The median TMB for men is 1.78 times that for women (Figure 2(a)).

Significantly higher TMB was observed in squamous carcinoma than that in adenocarcinoma (Figure 2(b)). The median TMB of lung adenocarcinoma is 4.3 Mutations/Mb, and the median TMB of lung squamous is 11.1 Mutations/Mb, 2.58 times that of lung adenocarcinoma (Figure 2(b)).

To investigate the association between TMB and age, we compared TMB (range, 0–52.2 Mutations/Mb; median, 5.3 Mutations/Mb) and patient age (range, 35–86 years; median, 63 years). Correlation analysis showed that the correlation between the two was not significant (correlation coefficient R = 0.160, P = 0.074) (Figure 2(c)).

### Analysis of most commonly mutated genes in patients with NSCLC

**Figure 3.**
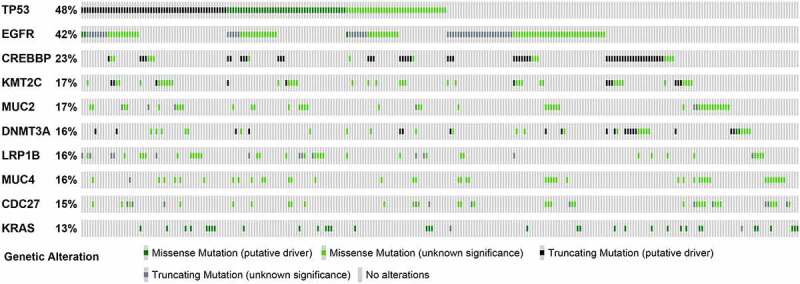
The top 10 high-frequency mutation genes of 206 patients were visualized by OncoPrinter. Each gray box from left to right represents the mutation of a sample

**Figure 4.**
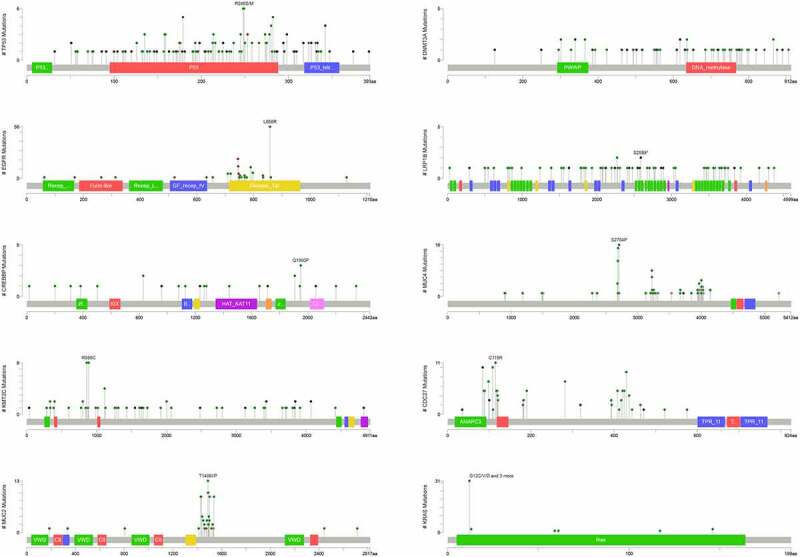
Diagram showing mutant sites and frequency of the top 10 genes harboring high-frequency mutations

Gene mutation has been proved to be closely associated with tumor development, and identification of the isoform of gene mutation might benefit therapy. We analyzed the ten most frequently mutated genes in tumor tissues of patients with NSCLC and found that all patients had at least one high-frequency mutation. Of the 206 cases, no KRAS mutation was observed in patients with EGFR mutations (Figure 3). MutationMapper analysis showed that, in addition to DNMT3A, the mutation sites of the other nine high-frequency mutation genes were R249S/M, L858R, Q1950P, R886 C, T1488I/P, S2589, S2704P, C115R, G12 CN/D. Out of these nine, mutant hotspots of TP53 (R249S/M), EGFR (L858R), and KRAS (G12 CN/D) were located P53 DNA-binding domain, Protein tyrosine kinase domain and Ras family domain respectively (Figure 4).

**Figure 5.**
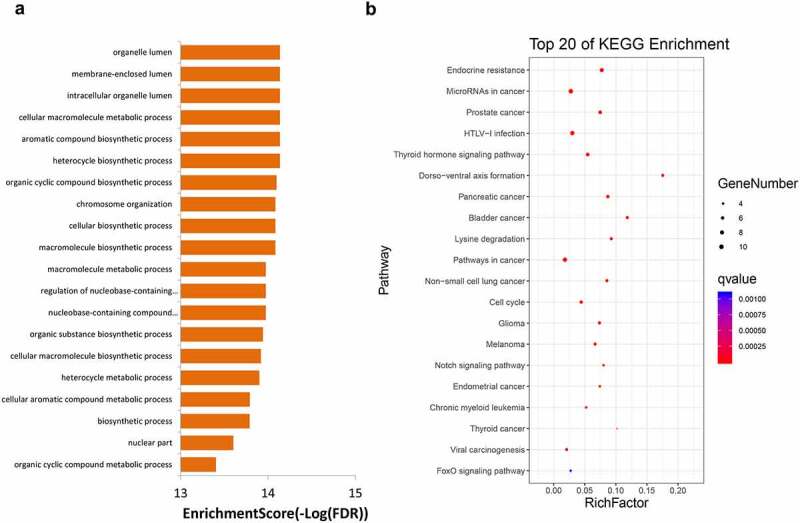
GO (a) and KEGG (b) enrichment analyses for genes with mutation frequency of >5%. GO, Gene Ontology, KEGG, Kyoto Encyclopedia of Genes and Genomes

GO and KEGG enrichment analyses showed that the top 10 high-frequency mutant genes were mainly enriched in terms of organelle lumen, membrane-enclosed lumen, intracellular organelle lumen, cellular macromolecule metabolic process, aromatic compound biosynthetic process (Figure 5(a)), and pathways including microRNAs in cancer, pathway in cancer, Notch signaling pathway, and FoxO signaling pathway (Figure 5(b)).

### Analysis of copy number variations in patients with NSCLC

**Figure 6.**
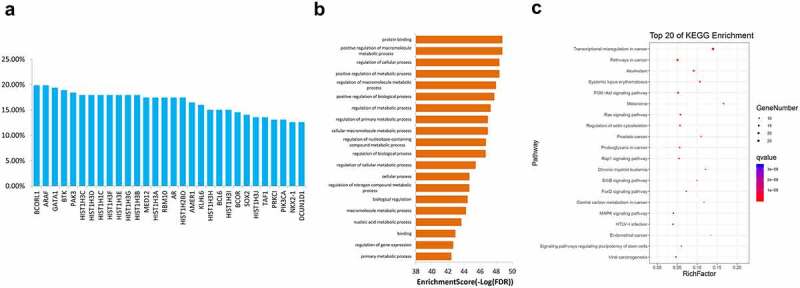
Of enrichment analysis of the 30 genes (a) and their GO (b) and KEGG enrichment analysis results (c) with the highest copy number increase in 206 samples. GO, Gene Ontology, KEGG, Kyoto Encyclopedia of Genes and Genomes

Because CNV may indicate dysregulated gene and protein expression that may ultimately affect development and progression of NSCLC, we further explored gene CNV in Chinese patients with NSCLC. CNV analysis showed that 110 genes had copy number amplification. Among these, BCORL1, ARAF, GATA binding protein 1 (GATA1), bruton tyrosine kinase (BTK), and P21 (RAC1) activated kinase 3 (PAK3) were the genes with the highest copy number amplification (Figure 6(a)). These genes are mainly concentrated in the terms of protein binding, positive regulation of macromolecule metabolic process, regulation of cellular process, positive regulation of metabolic process, and regulation of macromolecule metabolic process (Figure 6(b)). KEGG analysis revealed that, for the genes with the highest copy number amplification, their predicted functions were enriched in transcriptional dysregulation in cancer, pathway in cancer, PI3K-Akt signaling pathway, and Ras signaling pathway (Figure 6(c)).

**Figure 7.**
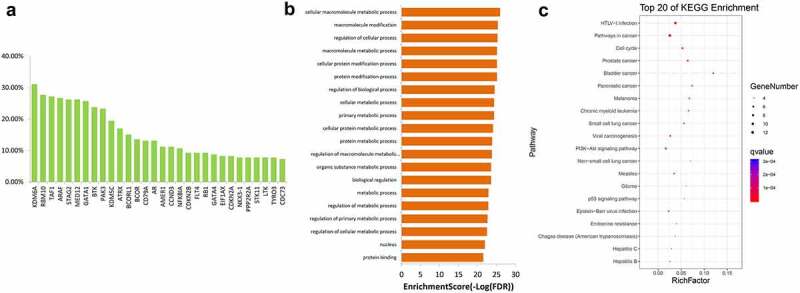
The top 30 genes with the highest copy number deletion in 206 samples (a) and their GO (b) and KEGG (c) enrichment analysis results. GO, Gene Ontology, KEGG, Kyoto Encyclopedia of Genes and Genomes

A total of 54 genes had copy number deletion. The genes with the highest copy number deletions were KDM6A, RBM10, TATA-box binding protein associated factor 1 (TAF1), ARAF, and stromal antigen 2 (STAG2) (Figure 7(a)). They were predicted to be enriched in terms of cellular macromolecule metabolic process, macromolecule modification, regulation of cellular process, macromolecule metabolic process, and cellular protein modification process (Figure 7(b)). The most enriched pathways were pathway in cancer, PI3K-Akt signaling pathway, and cell cycle (Figure 7(c)).

## Discussion

The purpose of this study was to identify the mutational characteristics of 206 Chinese patients with NSCLC. We identified 18,749 mutations by using targeted NGS. Among these mutations, missense mutations were dominant. Base mutations were dominated by pyrimidine and purine conversions. The ten most frequently mutated genes were obtained. Notably, EGFR and KRAS mutations were mutually exclusive. There were differences in TMB between the sexes and pathological subtypes; however, TMB was not associated with age. Finally, 110 genes and 54 genes showed copy number amplification and copy number deletion, respectively. These genes were specifically enriched in the NSCLC-associated pathways.

Based on the targeted NGS, we determined the most frequently mutated genes in Chinese patients with NSCLC. These genes were TP53, EGFR, CREBBP, KMT2C, MUC2, DNMT3A, LRP1B, MUC4, CDC27, and KRAS. Mutations in these genes have been reported previously in NSCLC [20]. Interestingly, the genes with the highest mutation frequency differed in their rankings compared with the findings of a study on the American population. In the study, they showed that the most frequently mutated gene in this report is KRAS, followed by EGFR [10]. However, our results are also consistent with the results in some reports. For example, a study in Lebanon showed that mutations of TP53 are common molecular changes, occurring in over 50% of tumors [21,22]. In an NSCLC study with a small sample size, TP53 was also found to be the most frequently mutated gene in the Chinese population [15]. These indicate that TP53 mutation might be one of the genes affected in Chinese patients with NSCLC. In addition, our results also support the idea reported in a previous study that the mutant hotspot area of TP53 is located in the common R249 area [23]. It has been accepted that TP53 is an important tumor suppressor and the most commonly mutated gene in most cancers. As a prognostic factor in NSCLC, the presence of TP53 mutation suggested an aggressive feature and poor clinical outcome [24].

Our results show that EGFR ranks second in terms of the mutation frequency, at a rate of 41.7%. Based on previous studies, the mutation rate of EGFR in Chinese patients with NSCLC is approximately 30%–50% [23,25]. The frequency of EGFR mutations that we obtained is also in this . It is worth mentioning that we found the hotspot mutation L858R of the EGFR gene, which is also considered to be a high-frequency mutation in Asia [26,27]. There is evidence that patients harboring common EGFR mutations exhibit approximately 10 months progression free survival time after EGFR tyrosine kinase inhibitor (TKI) therapy, whereas those with uncommon EGFR mutations exhibit less response to EGFR TKI [28–30]. Therefore, our findings indicate that most Chinese patients with NSCLC might benefit from EGFR TKI treatment. However, in those NSCLC harboring dual TP53/EGFR mutations, especially missense mutations, low response is frequently observed [31]. In addition to TP53 and EGFR, KRAS is also a commonly mutated gene in NSCLC. In some reports, it is described that the frequency of conversion of KRAS in the Chinese is approximately 8% [25,32]. Here, we report a mutation rate of the KRAS gene of 12.8% [33].

In contrast to the widely reported high-frequency mutated genes mentioned above, CREBBP (23.1%), KMT2C (16.9%), MUC2 (16.6%), DNMT3A (15.5%), LRP1B (15.5%), MUC4 (15.5%), and CDC27 (15.2%) are currently reported less in the Chinese population, although mutations in DNMT3A and KMT2C have been identified in some studies [20,33–35]. Our results suggest some aspects of the mutational characteristics of these genes in Chinese NSCLC, suggesting functions of these genes in the etiology and treatment of NSCLC. It is worth mentioning that we observed that patients with NSCLC having EGFR mutations have no KRAS mutations, and vice versa. This is consistent with the previous assertion that EGFR and KRAS mutations are mutually exclusive in NSCLC, although some cases of EGFR and KRAS mutations being present together in some Asian populations, including in China, have been reported [25,36].

The genome in NSCLC is unstable and exhibits a wide range of gene CNVs. Because CNV is closely related to the expression of mRNA and protein, copy number amplification or deletion may affect the expression of tumor-related genes and the patient’s sensitivity to treatment and survival [37]. Analysis of the variation of copy number is helpful for learning underlying mechanisms and functions of related genes in patients with NSCLC. Our results show that the genes with the most increased copy number were BCORL1, ARAF, and GATA1, while those with the greatest deletion of copy number were KDM6A, RBM10, TAF1, ARAF, and STAG2. Among these genes, evidence suggests that patients with high expression of BCORL1 have a shorter 3-year survival than patients with its low expression [38]. In addition, RBM10 functions to inhibiting the proliferation of non-adenocarcinoma cells [39]. We speculate that the increase in BCORL1 copy number and deletion of RBM10 copy number may suggest their roles in the pathogenesis of NSCLC.

The results of GO and KEGG enrichment analyses of genes with frequent mutations and CNV suggest that the mutant genes are enriched in tumor-related terms and signaling pathways. These pathways include the PI3K-Akt signaling pathway, FoxO signaling pathway, and Ras signaling pathway. The correlation between activation of the Notch signaling pathway and poor prognosis of NSCLC has been confirmed [40,41]. PI3K-Akt is an important signaling pathway that regulates tumor formation, survival and metastasis [42,43]. One of its downstream factors is the FoxO signaling pathway. Akt promotes the phosphorylation of FoxO and inhibits the transcriptional function of FoxO, potentially resulting in the induction of apoptosis, which is involved in biological processes such as NSCLC radiosensitization and tumor growth inhibition [44–46]. Moreover, the Ras signaling pathway is a proto-cancer pathway. Multiple tumor-promoting factors and drugs have been found to modulate tumor progression through this pathway [47–49]. Based on KEGG analysis, we suggest that the high frequency of mutation genes and CNV genes are associated with these tumor-related pathways. Inhibitors targeting these pathways may thus have clinical significance.

It is interesting to find that TMB was higher in men than in women. Since we were unable to correlate the current data such as TMB with the treatment outcomes of men and women, the clinical prognostic value of genetic mutations could not be derived. Subsequent research on the links between the mutant genes and the clinical data of this patient population will further enrich the clinical value of the mutant gene.

## Conclusion

The most common gene mutations in Chinese patients with NSCLC are missense mutations, and TP53, EGFR, CREBBP, KMT2C, MUC2 genes are the most frequently mutated genes. Several genes exhibited copy number amplification and copy number deletion. There were differences in TMB between the sexes and pathological subtypes; however, TMB was not associated with age. Our findings indicate that the panel is a good method for tumor molecular characterization In addition, our results are expected to provide clues for interpreting the etiology of NSCLC and performing drug target screening for this condition.

## Highlights

The TP53 gene occurs with the highest frequency in 206 Chinese patients with NSCLC.

TMB is higher in males than in females.

EGFR and KRAS mutations are mutually exclusive.

Genes with copy number variations are enriched in cancer-associated pathways.
